# Supplementary material for: N-Acetylcysteine Attenuates Sepsis-Induced Muscle Atrophy by Downregulating Endoplasmic Reticulum Stress
Source: Biomedicines. 2024 Apr 18;12(4):902. doi: 10.3390/biomedicines12040902 (PMC11048408; doi:10.3390/biomedicines12040902)
Supplement: Supplementary file 1 [file biomedicines-12-00902-s001.zip › biomedicines-2935473-supplementary.pdf]

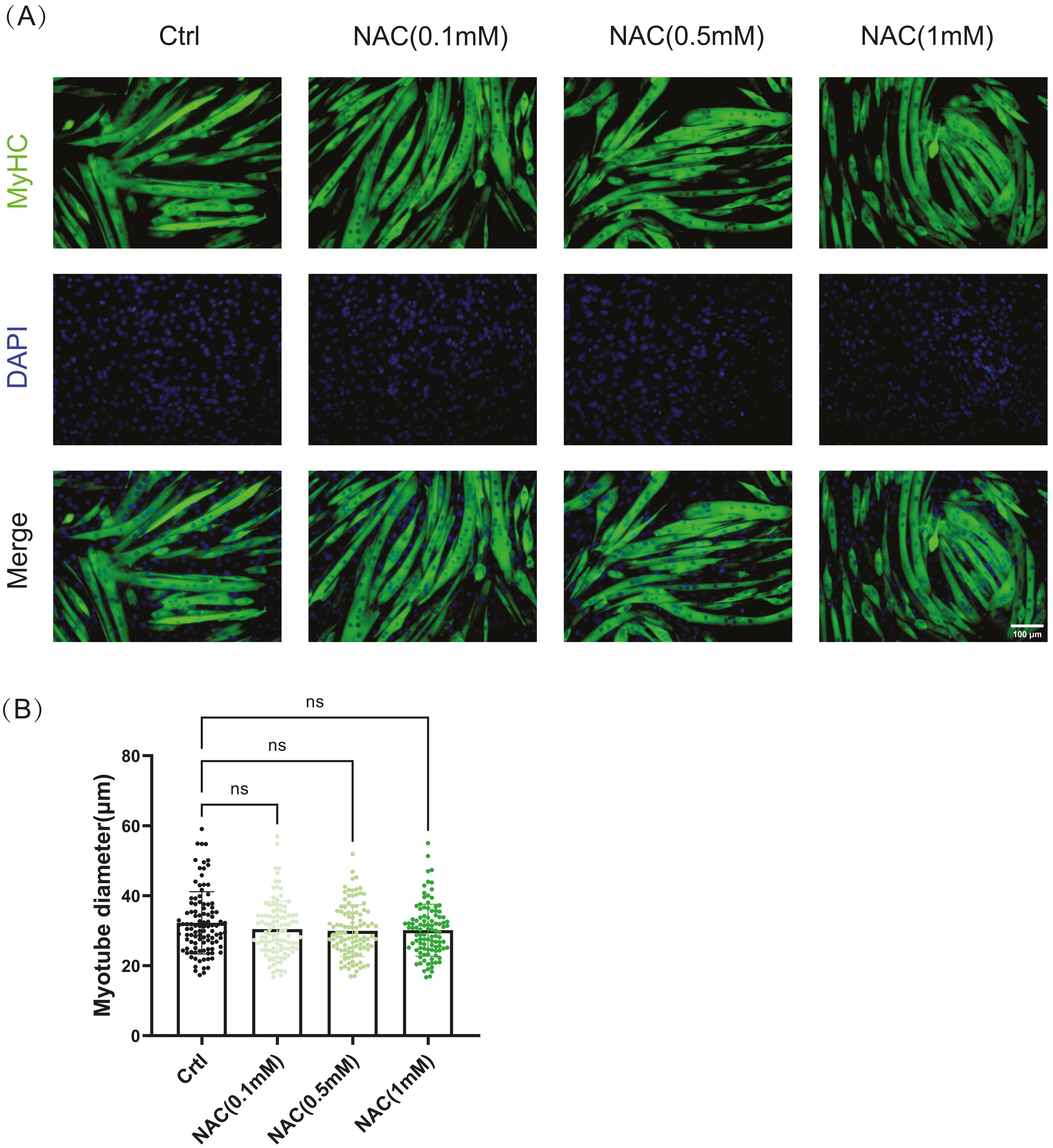

Supplemental Figure S1. Effect of NAC on myotube diameter. (**A**) Representative immunofluorescent images of myotubes treated with different concentrations of NAC. (**B**) Measurement of myotube diameter after administration of NAC. Scale bar = 100 μm.
